# 14-3-3τ Regulates Beclin 1 and Is Required for Autophagy

**DOI:** 10.1371/journal.pone.0010409

**Published:** 2010-04-29

**Authors:** Bing Wang, Shiyun Ling, Weei-Chin Lin

**Affiliations:** 1 Division of Hematology and Oncology, Department of Medicine, University of Alabama at Birmingham, Birmingham, Alabama, United States of America; 2 Department of Cell Biology, University of Alabama at Birmingham, Birmingham, Alabama, United States of America; University of Hong Kong, Hong Kong

## Abstract

**Background:**

Beclin 1 plays an essential role in autophagy; however, the regulation of Beclin 1 expression remains largely unexplored. An earlier ChIP-on-chip study suggested Beclin 1 could be an E2F target. Previously, we also reported that 14-3-3τ regulates E2F1 stability, and is required for the expression of several E2F1 target genes. 14-3-3 proteins mediate many cellular signaling processes, but its role in autophagy has not been investigated. We hypothesize that 14-3-3τ could regulate Beclin 1 expression through E2F1 and thus regulate autophagy.

**Methods and Findings:**

Using the RNAi technique we demonstrate a novel role for one of 14-3-3 isoforms, 14-3-3τ, in the regulation of Beclin 1 expression and autophagy. Depletion of 14-3-3τ inhibits the expression of Beclin 1 in many different cell lines; whereas, upregulation of 14-3-3τ induces Beclin 1. The regulation is physiologically relevant as an extracellular matrix protein tenascin-C, a known 14-3-3τ inducer, can induce Beclin 1 through 14-3-3τ. Moreover, rapamycin-induced, serum free-induced and amino acid starvation-induced autophagy depends on 14-3-3τ. We also show the expression of Beclin 1 depends on E2F, and E2F can transactivate the Beclin 1 promoter in a promoter reporter assay. Upregulation of Beclin 1 by 14-3-3τ requires E2F1. Depletion of E2F1, like 14-3-3τ, also inhibits autophagy.

**Conclusion:**

Taken together, this study uncovers a role for 14-3-3τ in Beclin 1 and autophagy regulation probably through regulation of E2F1.

## Introduction

Autophagy is an essential process for the lysosomal turnover of cellular macromolecules and organelles. Deregulation of autophagy has been linked to many pathological conditions, such as cancer. A link between autophagy and cancer has been appreciated through the observation that Beclin 1, a phylogenically conserved protein essential for autophagy, is a haplo-insufficient tumor suppressor [1]. Beclin 1 is part of the PI(3) kinase class III (PI(3)KC3) lipid kinase complex that plays a central role in the induction of autophagy [2]. Beclin 1 was originally identified as a protein binding to antiapoptotic Bcl-2 protein [3]. In addition to the activity in inhibiting apoptosis, Bcl-2 also inhibits autophagy through its ability in binding to the Beclin 1-PI(3)KC3 complex, an example of the interaction between two major pathways of cell death [4].

Altered levels of Beclin 1 are seen in many pathological conditions. Niemann-Pick C (NPC) disease is an autosomal recessive lipid storage disorder which is often caused by loss of function mutations in the *NPC1* gene. NPC1-deficient cells showed increased autophagy due to abnormally high levels of Beclin 1 [5]. Beclin 1 is induced at the site of traumatic brain injury and may serve as a mechanism to dispose injured cells through autophagy [6]. Autophagy is also induced by ischemia and further enhanced by reperfusion in the heart. In the reperfusion phase of the ischemia-reperfusion, Beclin 1 is induced and the elevated level of Beclin 1 is responsible for autophagy induction since the autophagy is significantly attenuated in *Beclin 1^+/−^* mice [7]. Thus, regulation of Beclin 1 appears to be an important mechanism to control autophagy under physiological and pathological conditions.

The tumor development in *Beclin 1^+/−^* mice indicates the importance of a full level of Beclin 1 expression for its tumor suppressor function [1]. Decreased levels of Beclin 1 are also seen in various cancer cells [8], [9]. Moreover, reduced levels of Beclin 1 protein have been correlated to poor patient outcome in many tumor types, such as esophageal cancer [10], stage III colon cancer [11], hepatocellular carcinoma [12] and high-grade glioma [13]. Therefore, abnormal levels of Beclin 1 expression may have adverse consequences.

14-3-3 proteins are a family of about 30kD dimeric well-conserved α-helical phosphoserine/threonine binding proteins. They contain seven mammalian isoforms (β,ε,γ,η,σ,τ,ζ), and are able to bind multiple protein ligands. The 14-3-3 binding proteins are very diverse; therefore, 14-3-3 is involved in many different cellular processes, including mitogenesis, DNA damage checkpoint, cell cycle control, and apoptosis [14]. Previously we showed that 14-3-3τ binds to ATM-phosphorylated E2F1 during DNA damage and promotes E2F1 stability, leading to induction of E2F1 pro-apoptotic target genes such as p73, Apaf1 and caspases [15]. In this report, we investigate a role for 14-3-3τ in the expression of a putative E2F target gene involved in autophagy, Beclin 1, and uncover its critical function in the regulation of autophagy induction.

## Results

**Figure 1:**
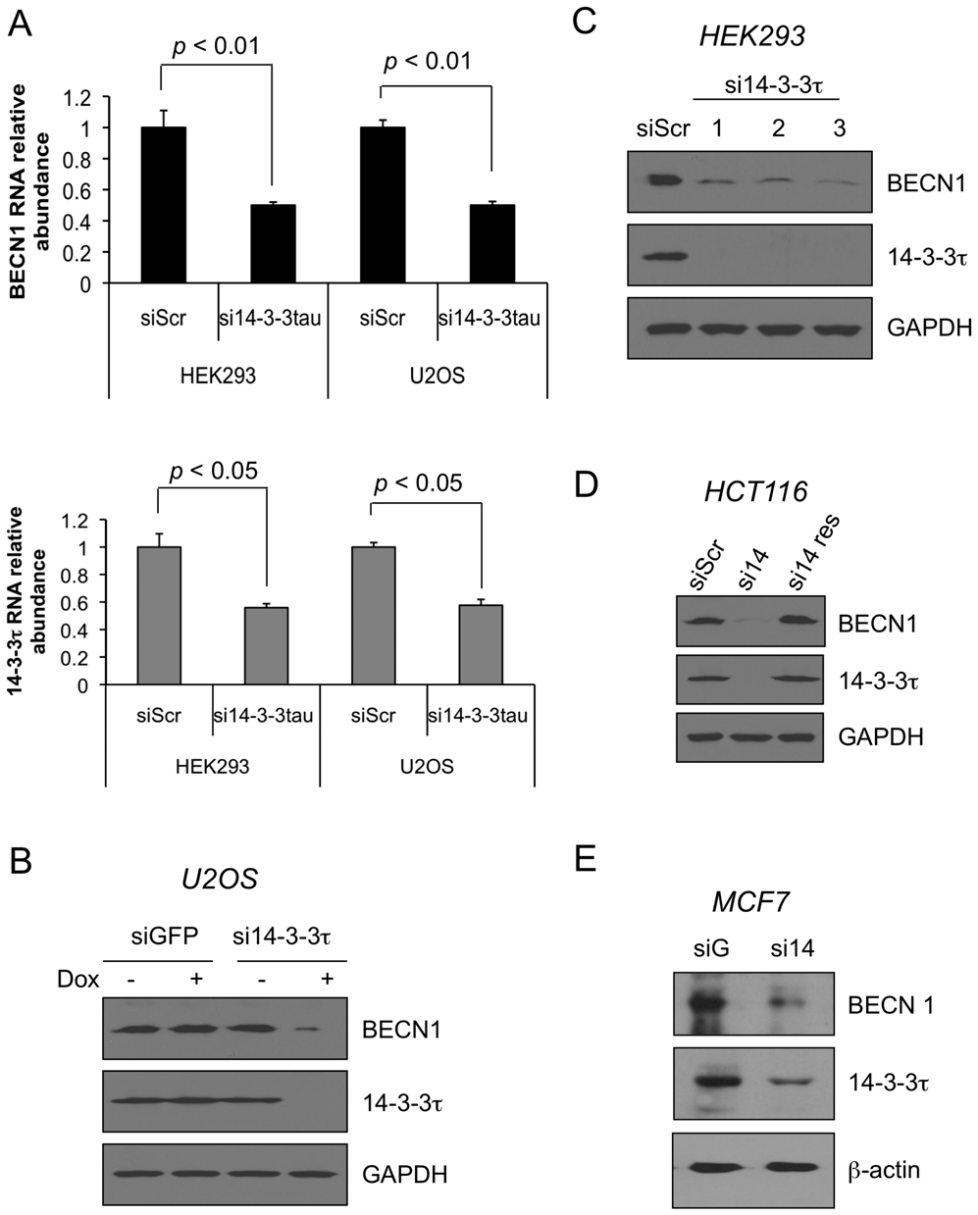
14-3-3τ is required for the expression of Beclin 1. (A) HEK293 and U2OS cells were transiently transfected with pSUPER-si14-3-3τ [15] or pSUPER expressing a scrambled sequence (siScr) [37]. Forty-four hr later, cells were harvested and RNA was extracted. Real-time RT-PCR analysis was performed using primers specific for Beclin 1, 14-3-3τ and GAPDH. The levels of Beclin 1 and 14-3-3τ were normalized to GAPDH levels and are expressed relative to the expression of the gene in the siScr control. The data shown represent the means ± standard deviations of triplicate samples. The *p* values are based on a paired two-tailed *t* test. (B) We established stable U2OS cell lines expressing an inducible siRNA against 14-3-3τ or a control GFP sequence under the control of tetracycline operon in pSUPERIOR.puro vector (OligoEngine). 14-3-3τ siRNA or a GFP siRNA was induced by doxycycline (DOX) (1 µg/ml), and cells were harvested for Western blot analysis using anti-Beclin 1, anti-14-3-3τ or anti-GAPDH antibody. (C) HEK293 cells were transiently transfected by three different pSUPER-14-3-3τ siRNA constructs (20 µg) by a calcium phosphate method. The cell lysates were harvested 48 hr later and analyzed by Western blot analysis. (D) HCT116 cells were transfected with 20 µg pSUPER-scrambled siRNA (siS) or si14-3-3τ (si14) along with a pcDNA3 empty vector or a vector expressing siRNA-resistant 14-3-3τ (res) by electroporation. The cell lysates were harvested 44 hr later and subject to Western blot analysis as indicated. (E) MCF7 cells were infected with a recombinant adenovirus expressing a control siGFP or si14-3-3τ (si14) at MOI of 100. Forty-two hr later, cells were harvested for Western blot analysis as indicated.

To investigate the role of 14-3-3τ in the control of gene expression, we established U2OS cell lines stably expressing inducible siRNA against either 14-3-3τ or a control GFP sequence under the control of tetracycline operon in pSUPERIOR.puro vector [16]. Using these cell lines, we performed a pilot microarray study and observed significant decrease (around 7-fold) of the Beclin 1 transcript expression upon depletion of 14-3-3τ. We confirmed the effect in two independent cell lines, HEK293 and U2OS cells, which were transiently transfected by an siRNA against 14-3-3τ or a scrambled sequence. The RNA was harvested, and the transcript levels of Beclin 1 and 14-3-3τ were measured by real-time RT-PCR (Fig. 1A). In both cell lines, depletion of 14-3-3τ led to reduced levels of Beclin 1. The change on Beclin 1 protein level upon addition of doxycycline to induce the expression of 14-3-3τ siRNA was also confirmed by Western blot analysis (Fig. 1B). The effect on Beclin 1 expression is truly due to depletion of 14-3-3τ since three different 14-3-3τ siRNAs all inhibited Beclin 1 expression (Fig. 1C), and Beclin 1 was restored to its baseline level when 14-3-3τ depletion was rescued with an RNAi-resistant 14-3-3τ construct (Fig. 1D). This effect was observed in multiple cell lines including U2OS (Fig. 1A–B), HEK293 (Fig. 1A & C), a colon cancer cell line HCT116 (Fig. 1D) and a breast cancer cell line MCF7 (Fig. 1E), suggesting that regulation of Beclin 1 by 14-3-3τ is general rather than cell line specific.

**Figure 2:**
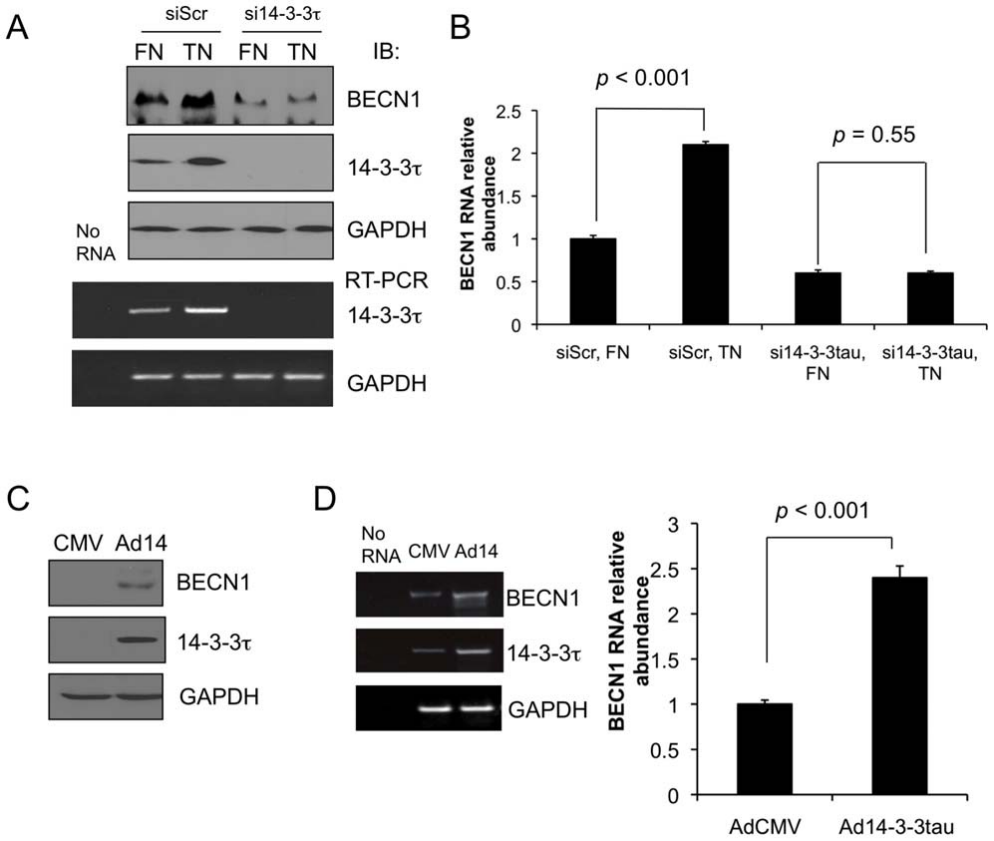
Tenascin-C upregulates Beclin 1 through 14-3-3τ. (A–B) MCF7 cells were grown on Fibronectin (FN) or Tenascin C (TN) pre-treated plates. Next day, cells were infected with Adsi14-3-3τ or control AdsiScr virus at MOI 100 for 48 hrs. Cells were then harvested for Western blot (A, upper panels), semiquantitative RT-PCR (A, lower panels), and real-time RT-qPCR analyses (B). No RNA lane represents a control RT-PCR reaction without input RNA. Quantitative PCR was performed in triplicate. The levels of Beclin 1 were normalized to GAPDH levels and are expressed relative to the expression of the gene in the siScr FN control. The data shown represent the means ± standard deviations of three independent samples. The *p* values are based on a paired two-tailed *t* test. (C–D) U2OS cells were infected by Ad14-3-3τ (Ad14) or an empty vector AdCMV (CMV) at MOI 300 for 2 days. Protein and RNA were harvested for Western blot analysis (C) or real-time RT-qPCR (D). Quantitative PCR was performed in triplicate. The levels of Beclin 1 were normalized to GAPDH levels and expressed relative to the expression of the gene in the AdCMV control. The data shown represent the means ± standard deviations of three independent samples. The *p* values are based on a paired two-tailed *t* test.

To investigate the regulation of Beclin 1 by 14-3-3τ in a physiological context, we made use of an extracellular matrix protein tenascin-C, which has been reported to induce 14-3-3τ in MCF7 cells when compared with a control fibronectin matrix protein [17]. Tenascin-C is an extracellular matrix component which is transiently expressed in association with epithelial cell detachment [18], proliferation, and migration. Extracellular matrix detachment has been recognized to be able to induce autophagy [19]. Recent studies also showed that the expression of tenascin-C in invasion borders of early breast cancer significantly correlates with proliferative activity, higher risk of distant metastasis and local recurrence [20]. Expression of tenascin-C was shown to be able to induce cell invasion through matrix metalloproteinase in breast cancer [21] and glioma cells [22]. The process of cell invasion can also induce autophagy [23]. We therefore hypothesized that tenascin-C might regulate Beclin 1 through 14-3-3τ. As shown in Figure 2A–B, tenascin-C induced 14-3-3τ and Beclin 1 when compared with fibronectin control, and the induction of Beclin 1 was blocked when 14-3-3τ was depleted. These results indicate that the induction of Beclin 1 by tenascin-C is mediated through 14-3-3τ. We want to point out that Beclin 1 expression was detectable both by immunoblotting and by qRT-PCR in our MCF7 cells, despite this cell line harbors 17q21 loss of heterozygosity for *Beclin 1* [24] and was reported to express undetectable levels of Beclin 1 [8], [25], [26]. It is unclear at this moment whether the expression of the remaining *Beclin 1* allele is activated in our MCF7 cells during passages or it is simply a matter of assay sensitivity. A role in the regulation of Beclin 1 for 14-3-3τ was also evaluated by overexpression. When 14-3-3τ was overexpressed in U2OS cells, both the transcript and protein of Beclin 1 were induced (Fig. 2C–D).

**Figure 3:**
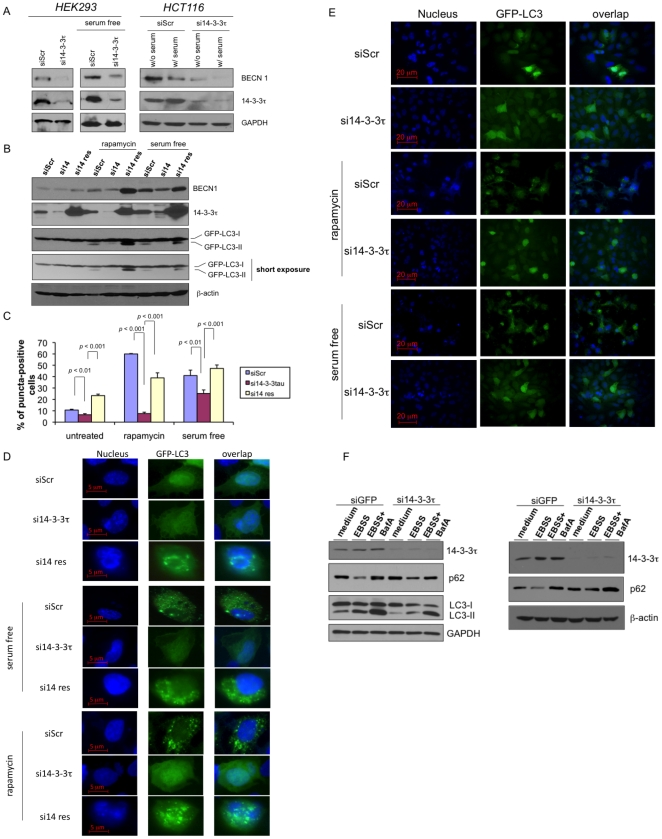
A role for 14-3-3τ in autophagy induction under nutrient limited conditions. (A) 14-3-3τ knockdown inhibits Beclin 1 protein expression in nutrient limited conditions. HEK293 or HCT116 cells were transfected with 20 µg pSUPER-si14-3-3τ or pSUPER-siScr. 24 hours later, cells were either left in nutrient-full medium (w/serum) or serum free medium (medium without supplement of serum) for 48 hours. Western blot analysis was performed using antibodies as indicated. (B–E) U2OS cells were transiently transfected with 5 µg GFP-LC3 by Lipofectamine. 24 hr later, the cells were infected with AdsiGFP or Adsi14-3-3τ (MOI 100) along with an empty vector AdCMV or a recombinant adenovirus expressing siRNA-resistant 14-3-3τ (res) (MOI 30). Next day, the cells were treated with 100 nM rapamycin for 24 hours or left in serum-free medium for 48 hours. (B) Some cells were harvested and subject to Western blot analysis as indicated. GFP-LC3 was probed with an anti-GFP antibody. (C–E) Cells were scored for GFP–LC3 autophagosomes-positivity as described in Materials and Methods. The graphs represent data derived from three independent experiments with means ± standard deviations. The *p* values are based on a paired two-tailed *t* test. (D): High magnification (1000×) images of representative cells. (E): Lower magnification (200×) images of representative fields. (F) Autophagy flux analysis. Left panel: The doxycline-inducible siRNA stable U2OS cell lines as described in Fig. 1B were first treated with doxycycline (1 µg/ml) for 7 days to deplete14-3-3τ. The cells were then washed with PBS for three times and then starved in Earle's balanced salt solution (EBSS) or EBSS with bafilomycin A1 (BafA) (0.1 µM) in 37°C for 2 hr before harvesting for Western blot analysis. Right panel: U2OS cells were infected with AdsiGFP or Adsi14-3-3τ at MOI 200. Three days later, the cells were washed with PBS and starved with EBSS or with EBSS + BafA as above for 3 hr. The cells were then harvested for Western blot analysis as indicated.

Depletion of 14-3-3τ also inhibited the expression of Beclin 1 during serum starvation or rapamycin treatment in either HEK293 cells, HCT116 cells or U2OS cells (Fig. 3A–B). Given a pivotal role for Beclin 1 in autophagy, we then investigated whether 14-3-3τ is required for autophay. During autophagy, LC3 (microtubule-associated protein-1 light chain-3) -I is lipidated to the LC3-II form, which integrates into the autophagosome membrane and form a distinctly punctate distribution [27]. We measured autophagy by detecting the appearance of LC3-II on Western blot analysis (Fig. 3B) or by the distinctly punctate distribution of GFP-LC3 under fluorescence microscope (Fig. 3D–E). Indeed, 14-3-3τ depletion significantly blocked autophagy induced by either serum starvation or by rapamycin treatment (Fig. 3B–E). When the depletion of 14-3-3τ was rescued by an RNAi-resistant construct, the expression of Beclin 1 increased and the autophagic response to serum starvation and rapamycin was also recovered (Fig. 3B–D). The relatively high level of autophagy in the “rescue” arm of the untreated group may be a result of overexpression of 14-3-3τ (Fig. 3B–C). To further substantiate a role for 14-3-3τ in autophagy induction, we also examined the autophagy induced by amino acid starvation by starving the cells in Earle's balanced salt solution (EBSS) and performed autophagy flux analysis (Fig. 3F). We first depleted 14-3-3τ in U2OS cells by doxycycline-inducible expression of si14-3-3τ (Fig. 3F, left panels) or by infecting U2OS cells with a recombinant adenovirus expressing si14-3-3τ (Fig. 3F, right panels). EBSS treatment induced autophagy as revealed by degradation of p62 and an increase of LC3-II. Comparing with siGFP control, 14-3-3τ depletion decreased the extent of p62 degradation and LC3-II formation (Fig. 3F). Upon treatment of the lysosomal inhibitor bafilomycin A1, which prevents the autophagic degradation of p62 and LC3-II, both p62 and LC3-II levels increase. The difference in p62 level between cells treated with bafilomycin A1 and untreated cells represents the amount of p62 that has been recruited into autophagic vesicles during the period of treatment. As shown in Fig. 3F, 14-3-3τ depletion inhibited the autophagic degradation of p62. Since bafilomycin A1 inhibits the degradation of autophagosomes, the levels of LC3-II in bafilomycin A1-treated cells reflect the rate of autophagosome formation (autophagy flux). By this measure, depletion of 14-3-3τ also decreased the autophagosome synthesis (Fig. 3F, left panels). Thus, combining the GFP-LC3 punctate, p62 degradation and autophagy flux analyses, we conclude that 14-3-3τ is required for nutrient deprivation- or rapamycin-induced autophagy.

**Figure 4:**
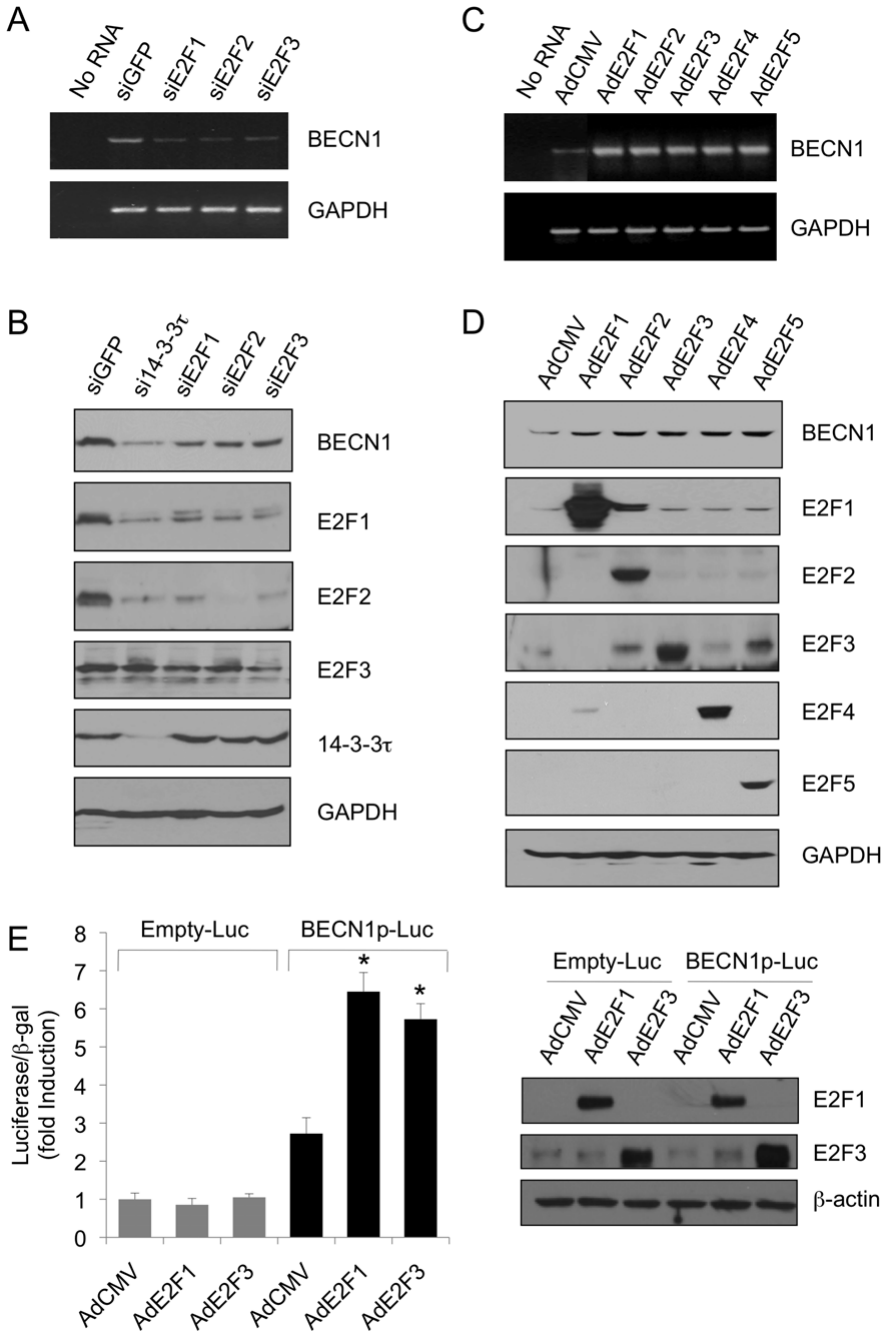
E2Fs regulate Beclin 1. (A–B) In addition to the doxycycline-inducible si14-3-3τ stable cell line as described in figure 1B, we also established stable U2OS cell lines expressing an inducible siRNA against E2F1, E2F2 or E2F3 [36] under the control of tetracycline operon in the pSUPERIOR.puro vector. 14-3-3τ siRNA, E2F siRNA or GFP siRNA was induced by doxycycline treatment (1 µg/ml) and cells were harvested for RNA extraction/RT-PCR assays (A), and for Western blot analysis (B). No RNA lane represents a control RT-PCR reaction without input RNA. (C–D) T98G cells were serum-starved for 48 hours and then infected by AdE2F1, AdE2F2, AdE2F3, AdE2F4 or AdE2F5 at MOI 200 for 24 hours. Cell lysates were then analyzed by RT-PCR assays (C) or Western blot analysis (D). (E) The activities of E2F1 and E2F3 toward the Beclin 1 promoter were tested in T98G cells using a human Beclin 1 promoter-Luciferase (BECN1p-Luc) reporter construct as described in the Materials and Methods. (*) *P*<0.05 (paired two-tailed *t* test) compared with AdCMV control infection in BECN1p-Luc transfected cells. Right panels: a portion of the cellular lysates was immunoblotted with antibody for E2F1, E2F3 or β-actin.

When Weinmann and colleagues performed chromatin immunoprecipitation to clone the E2F-bound chromatins, Beclin 1 promoter was identified to be one of the promoters occupied by many E2F family members [28]. This observation suggests that *Beclin 1* is an E2F target gene. Our prior study showed that 14-3-3τ regulates E2F1 stability and as such regulates many E2F1 target genes [15]. Therefore, we investigated whether Beclin 1 is regulated by E2F. Indeed, the expression of Beclin 1 also depends on E2F. When E2F1, or 2 or 3 was depleted, Beclin 1 RNA (Fig. 4A) and protein (Fig. 4B) levels were significantly reduced in U2OS cells. Due to the fact that E2F1, 2 and 3 regulates each other, depletion of one E2F also affected to some extent the expression of the others. When E2F1, or 2 or 3 or 4 or 5 was overexpressed in serum-starved T98G cells, Beclin 1 RNA (Fig. 4C) and protein (Fig. 4D) was induced. To demonstrate that E2Fs can directly transactivate the Beclin 1 promoter, we performed Beclin 1 promoter reporter assay in T98G cells. As shown in Fig. 4E, both E2F1 and E2F3 trans-activated the Beclin 1 promoter activity. Taken together, these data confirmed that *Beclin 1* is an E2F target gene.

**Figure 5:**
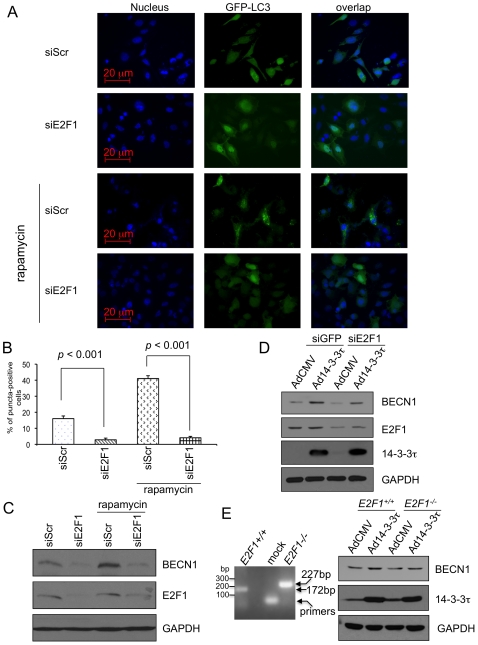
A role for E2F1 in autophagy and the regulation of Beclin 1 by 14-3-3τ. (A–C) HeLa cells were transiently transfected with 3 µg GFP-LC3 and 20 µg pSUPER-siE2F1 or pSUPER-siScr using Lipofectamine 2000. 24 hr later, the cells treated with 100 nM rapamycin for 24 hr to induce autophagy. (A) Cells were fixed in 1% paraformaldehyde, and nuclei were stained with Hoechst 33258. Images were taken with 200× magnification in a Zeiss Axioplan 2 digital fluorescence microscope. (B) More than 500 GFP-positive cells per sample were scored for GFP-LC3 puncta. The data are derived from three independent experiments. The data shown represent the means ± standard deviations. The *p* values are based on a paired two-tailed *t* test. (C) Some cells were harvested and the cellular lysates were subject to Western blot analysis as indicated. (D) The E2F1 siRNA or GFP siRNA in the U2OS cell lines as described in Fig. 4A–B was induced by doxycycline (1 µg/ml) for 3 days, and then the cells were infected with AdCMV or 14-3-3τ at MOI 300. 48 hr later, cells were harvested for Western blot analysis. (E) Primary *E2F1^+/+^* or *E2F1^−/−^* mouse embryonic fibroblasts (MEFs) were infected with AdCMV or 14-3-3τ at MOI 1000. 48 hr later, cells were harvested for Western blot analysis (Right panel). Left panel: Genotyping of the MEFs. The 172-bp and 227-bp products were amplified from the wild type and mutant alleles respectively. Mock represents no template input control.

Finally, we investigated the role for E2F1 in autophagy induction and in the regulation of Beclin 1 by 14-3-3τ. Depletion of E2F1 significantly abrogated rapamycin-induced autophagy (Fig. 5A–C). This result is consistent with the finding that E2F1 is required for etoposide-induced autophagy [29]. Depletion of E2F1 or deletion of *E2F1* gene also affected the accumulation of Beclin 1 during overexpression of 14-3-3τ (Fig. 5D–E). Taken together, these data provide evidence for a link from 14-3-3τ to autophagy regulation through the control of E2F1-Beclin 1.

## Discussion

To our knowledge, this study presents the first evidence supporting a critical role for 14-3-3 proteins in autophagy. We also elucidate its mechanism. We demonstrate that 14-3-3τ regulates the expression of Beclin 1 through E2F1 and provide evidence for its role in autophagy induction during nutrient deprivation or rapamycin treatment. We also show that E2Fs can directly regulate the Beclin 1 promoter and expression. Previously, we showed that 14-3-3τ controls E2F1 stability [15]. Thus, the role for 14-3-3τ in autophagy is likely related to its role in the regulation of E2F1 and as a result Beclin 1 expression. Indeed, E2F1 mediates the upregulation of Beclin 1 by 14-3-3τ. Since 14-3-3τ is involved in many signaling processes, e.g. ubiquitin-independent proteasomal degradation of p21 [30], we cannot rule out the possibility that 14-3-3τ may also be involved in other mechanisms for autophagy induction. Importantly, we showed that tenascin-C, an extracellular matrix protein involved in detachment (a process which causes autophagy [19]), induces Beclin 1 through 14-3-3τ, thus placing the tenascin-C/14-3-3τ/E2F1/Beclin 1 regulation in a physiological context.

In addition to Beclin 1, E2Fs also regulate many genes involved in autophagy. Previously, Macleod and colleagues identified BNIP3 as an E2F-regulated gene that is involved in hypoxia-induced autophagy [31]. Polager and coworkers also showed that E2F1 regulates several autophagy-related genes including LC3, autophagy-related gene-1 (ATG1) (also named UNC51-like kinase 1, ULK1), ATG5 and damage-regulated autophagy modulator (DRAM) [29]. The ability of E2F in inducing Beclin 1 expression (Fig. 4C–D) and the requirement of E2F1-3 for Beclin 1 (Fig. 4A–B) in conjunction with the observation that Beclin 1 promoter is occupied by E2F family members [28] strongly suggest that Beclin 1 is an E2F target as well. Indeed, we have confirmed this by showing the ability of E2F1 and E2F3 in transactivating Beclin 1 promoter by a reporter assay (Figure 4E). Since most E2F members occupy on the Beclin 1 promoter [28] and induce its expression (Fig. 4C–D), Beclin 1 appears to be regulated by multiple E2F members. Recently, Kusama at al. amplified and cloned the putative promoter regions of 23 human ATG genes to check their responsiveness to E2F1 in a promoter-luciferase reporter assay. Thirteen promoters were reported to be regulated by E2F1 in HeLa cells [32]. In addition to the known target genes, LC3 and DRAM, other E2F1-induced promoters are: ULK2, ATG4, ATG7, GABARAPL2, ATG9A, ATG10 and ATG12. The promoter of GABARAPL1 was down regulated by E2F1. Probably due to short promoter constructs for ATG1/ULK1 (nucleotide position −184/+6) and Beclin 1 (nucleotide position −207/+13) used in Kusama's study, both were not among these promoters. The Beclin 1 promoter construct used in our study comprises 949 bp (nucleotide position −856/+93). This suggests that the E2F-responsive sequences in the Beclin 1 promoter might be located within the −856/+93 region, but outside of the −207/+13 region. However, we cannot rule out the possibility that other elements of the promoter more upstream to −856 might be important for optimal expression of Beclin 1. A putative Beclin 1 promoter DNA fragment has been shown to have a high-affinity E2F in vivo binding, but interestingly does not contain a consensus E2F site [28]. Nevertheless, it does contain extremely GC-rich sequences, which is a hallmark of many E2F-regulated promoters. Recently, it was also reported that Beclin 1 promoter could be activated by NFκB [33]. A detailed analysis of the Beclin 1 promoter is warranted in the future.

Our results identify 14-3-3τ as an important regulator for Beclin 1 and autophagy. With the involvement of 14-3-3 in many important biological signaling processes, this regulation provides a potential link between these processes and autophagy. For example, tenascin-C signaling is involved in extracellular matrix detachment which can induce autophagy [19]. Through the induction of 14-3-3τ, tenascin-C can in turn up-regulates Beclin 1 expression (Fig. 2). This could contribute to the autophagy induced by cell invasion. It is desirable to further test the regulation in other physio/pathological contexts, for example, the induction of Beclin 1 and autophagy during reperfusion phase after cardiac ischemia. While our data show a role for 14-3-3τ in the expression of Beclin 1, and autophagy induction during nutrient depletion or rapamycin treatment, high expression of 14-3-3τ or Beclin1 alone may not induce autophagy. However, high levels of 14-3-3τ or Beclin 1 in certain physio/pathological conditions might prime cells for autophagy when the cells receive the autophagy stimuli. This possibility deserves future investigation.

## Materials and Methods

### Cell culture and transfection

HEK293, breast cancer cell line MCF7, cervical cancer cell line HeLa, and glioblastoma cell line T98G were grown in Dulbecco's modified Eagle's medium (DMEM) supplemented with 10% fetal bovine serum (FBS), penicillin (50 IU/ml), and streptomycin (50 µg/ml) in a humidified incubator with 5% CO_2_ at 37°C. Colon carcinoma cell line HCT116 and human osteosarcoma cell line U2OS were maintained in McCoy's 5A supplemented with 10% fetal bovine serum (FBS), penicillin (50 IU/ml), and streptomycin (50 µg/ml). MCF7 cell line is a gift from J. Michael Ruppert. All other cell lines were purchased from ATCC. Cells were transfected by the calcium phosphate method, Lipofectamine 2000 (Invitrogen) according to manufacturer's protocol, or the Gene Pulser Xcell electroporation system (Bio-Rad). The Gene Pulser Xcell electroporation conditions for HCT116 cells are: Voltage 155 V, Capacitance 1000 µF, Resistance ∝Ω, 20 µg DNA in 200 µl tranfection medium (McCoy's 5A without supplement) within 2 mm Bio-Rad electroporation cuvettes. Bafilomycin A1 was purchased from Fisher Scientific (Pittsburgh, PA). *E2F1^+/+^* and *E2F1^−/−^* primary mouse embryonic fibroblasts (MEFs) (passage 3) were prepared from 13.5-day-old embryos and grown in DMEM supplemented with 10% FBS as described [34], [35]. We followed the protocol described in Jackson Laboratory Genotyping Protocol# 002785, http://jaxmice.jax.org/protocolsdb for genotyping of the E2F1-knockout MEFs. Earle's balanced salt solution (EBSS) was purchased from Gibco-Invitrogen Co.

### Recombinant Plasmids and adenoviruses

The construction of pCMV-SPORT6-14-3-3τ, pSUPER-si14-3-3τ, pSUPER-siE2F1, pSUPER-siE2F2, pSUPER-siE2F3 has been described [15]. The 19-nt target sequences for si14-3-3τ #1: 5′-GGACTATCGGGAGAAAGTG-3′; si14-3-3τ#2: 5′- CGAGGAGCGCAACCTGCTC-3′; si14-3-3τ#3: 5′-GAACGTGGTCGGGGGCCGC-3′. To construct an siRNA-resistant expression vector and rescue the depletion by si14-3-3τ #1, the pCMV-SPORT6-14-3-3τ was used to generate a silent mutation by changing four nucleotides (5′-GCTGATTAAGGACTACAGAGAAAAAGTGGAGTCCGAG-3′) using the QuickChange site-directed mutagenesis kit (Stratagene). The mutation was verified by sequencing. To construct Adsi14-3-3τ and AdsiGFP, the NotI/SalI fragments of pSUPER constructs containing H1 RNA gene promoter and si14-3-3τ or siGFP were cloned to pShuttle followed by recombination with an AdEasy vector. Ad14-3-3τ was constructed by moving the siRNA-resistant 14-3-3τ cDNA from pCMV-SPORT6-14-3-3τ by XhoI/NotI to pShuttle-CMV followed by recombination with an AdEasy vector. All viruses were purified by a double CsCl banding procedure in a Beckman ultracentrifuge.

### Real-time RT-PCR

RNA was extracted using TRIzol reagent (Invitrogen). Equal amounts (2 µg) of RNA were used for first strand cDNA synthesis with reverse transcriptase. An equal portion of the mixture was used for PCR reactions to detect the expression of Beclin 1 and 14-3-3τ with the following primer pairs: Beclin 1, 5′-CTTACCACAGCCCAGGCGAAAC-3′, and 5′-GCCAGAGCATGGAGCAGCAA-3′; 14-3-3τ, 5′-TCCTGCACTGTCTGATGTCC-3′, and 5′-GGACTATCGGGAGAAAGTGG-3′ with annealing temperature set at 58°C (Beclin 1) or 62°C (14-3-3τ) for 25 cycles. For quantitative PCR, each sample was performed in triplicate on an MX3005P thermal cycler (Stratagene) using the SYBR green dye method to track the progress of the reactions with ROX dye added as reference. The qPCR program for Beclin1 is: 95°C 30 sec, 58°C 1 min and 72°C 1 min; the qPCR program for 14-3-3τ is: 95°C 30 sec, 62°C 1 min and 72°C 1 min. GAPDH was run in parallel with test genes. The primers for GAPDH had been reported [36]. Results were analyzed with MxPro 4.0 quantitative PCR software (Stratagene).

### GFP-LC3 punctates quantification

HeLa cells were transiently transfected with GFP-LC3 and siRNA constructs. 24 hr later, cells were then treated with 100 nM rapamycin for 24 hr. Cells were then fixed in 1% paraformaldehyde, and nuclei were stained with Hoechst 33258. Cells were examined by fluorescence microscopy. To quantify GFP–LC3 autophagosomes-positive cells, multiple random fields representing more than 500 GFP-positive cells were counted. Cells with five or more GFP-LC3 vacuoles (puncta) were considered positive for autophagy.

### Western blot analysis

The transfected cells were harvested in SDS lysis buffer (1% SDS, 60 mM Tris PH 6.8) and the lysates were boiled for 5 min followed by a brief sonication to homogenize the lysates. Equal amounts (100 µg) of protein lysates were fractionated by SDS-PAGE and electrotransferred to an Immobilon-P membrane (Millipore). Equal protein loading was confirmed with Ponceau-S staining. The specific signals were detected with appropriate antibodies. The antibodies specific to 14-3-3τ (C-17), BECN1 (E8), E2F1 (KH95), E2F2 (C20), E2F3 (C-18), E2F4 (WUF11), E2F5 (E-19), GST (B-14), GFP (B2) and GAPDH (0411) were purchased from Santa Cruz Biotechnology (Santa Cruz, CA). The monoclonal antibody for 14-3-3τ (3B9) and β-actin antibody (A2066) were purchased from Sigma (St Louis, MO). LC3 antibody (NB100-2220) was purchased from Novus Biologicals (Littleton, CO), and mouse monoclonal p62 antibody (clone 2C11, cat. number: H00008878-M01) was obtained from Abnova (Taipei, Taiwan). On average, the western blot analysis was performed two to three times to ensure the reproducibility of the data.

### Luciferase assay

Beclin 1-promoter-Luciferase reporter construct was obtained from Switchgear Genomics (product ID: S119414). This pGL-based vector construct contains a 949-bp human Beclin 1 promoter. An empty pGL3-Basic vector was used as a control. T98G cells were transfected using Lipofectamine 2000 reagent with promoter plasmid (5 µg) and pCMV-β-galactosidase plasmid (5 µg) and grown in DMEM with 10% FBS. Sixteen hr later, the media were replaced with serum-free DMEM and then cells were infected with a control AdCMV empty vector virus or AdE2F1 or AdE2F3 at MOI of 200. Cells were grown in serum-free media for additional 36 hr. Cells were harvested in PBS; an aliquot was lysed in SDS lysis buffer for Western blotting, while the rest of the sample was lysed in reporter lysis buffer (Promega). Luciferase activity and β-galactosidase activity were measured according to manufacturer instruction. Luciferase activity was normalized against β-galactosidase activity. All assays were performed in triplicate.

### Statistical analysis

The levels of Beclin 1 and 14-3-3τ were measured by quantitative RT-PCR and then normalized to GAPDH levels. The experiment was run in triplicate for each sample. The differences of the levels between samples were compared using paired two-tailed *t* test. P values less than 0.05 were considered statistically significant. GFP-LC3 punctate-positive cells were scored under fluorescence microscope. Each experiment was run in triplicate, and more than 500 GFP-positive cells were scored per slide. The differences of the percentages between samples were compared by paired two-tailed *t* test. Each sample in the Beclin 1 promoter luciferase reporter assay was also run in triplicate and the differences between samples were compared using paired two-tailed *t* test.
